# Prevalence and Risk Factors for Newborn Anemia in Southwestern Uganda: A Cross-Sectional Study

**DOI:** 10.1155/2024/5320330

**Published:** 2024-04-02

**Authors:** Joseph Ngonzi, Leevan Tibaijuka, Timothy Mwanje Kintu, Raymond Bernard Kihumuro, Onesmus Ahabwe, Onesmus Byamukama, Wasswa Salongo, Julian Adong, Adeline A. Boatin, Lisa M. Bebell

**Affiliations:** ^1^Department of Obstetrics and Gynecology, Faculty of Medicine, Mbarara University of Science and Technology, Mbarara, Uganda; ^2^Department of Paediatrics and Child Health, Faculty of Medicine, Mbarara University of Science and Technology, Mbarara, Uganda; ^3^Department of Obstetrics and Gynecology, Massachusetts General Hospital, Boston, USA; ^4^Harvard Medical School, Boston, USA; ^5^Harvard Medical School, Department of Medicine, Center for Global Health and Medical Practice Evaluation Center, Massachusetts General Hospital, Boston, USA

## Abstract

**Introduction:**

The global prevalence of maternal anemia is about 42%, and in sub-Saharan Africa, the prevalence of newborn anemia ranges from 25% to 30%. Anemia in newborn babies may cause complications such as delayed brain maturation and arrested growth. However, there is limited data on the prevalence of newborn anemia and its risk factors in people living in resource-limited settings.

**Objectives:**

We determined the prevalence and risk factors for newborn anemia and its correlation with maternal anemia in southwestern Uganda.

**Methods:**

This was a cross sectional study of 352 pregnant women presenting to the Mbarara Regional Referral Hospital for delivery. We collected maternal blood in labor and umbilical cord blood from the placental vein. We measured hemoglobin using a point-of-care Hemocue machine. We used summary statistics to characterize the study participants and compared demographic characteristics and outcomes using chi-square, *t*-test, and Wilcoxon rank sum analyses. We defined newborn anemia as umbilical cord hemoglobin <13 g/dl and measured the relationship between maternal and umbilical cord hemoglobin using linear regression analysis.

**Results:**

The prevalence of newborn anemia was 17%. Maternal parity was significantly higher for anemic than nonanemic newborns (3 versus 2, *P*=0.01). The mean age in years (SD) was significantly lower for participants with umbilical cord hemoglobin <13 g/dl than those ≥13 g/dl (26 years [5.6] versus 28 [6.3], *P*=0.01). In multivariable linear regression analysis, a 1-point decrease in maternal hemoglobin was associated with a 0.14-point decrease in umbilical cord hemoglobin (*P*=0.02). Each one-unit increase in parity was associated with a 0.25-point decrease in umbilical cord hemoglobin (*P*=0.01). Cesarean delivery was associated with a 0.46-point lower umbilical cord hemoglobin level compared with vaginal delivery (*P*=0.03).

**Conclusions:**

We found a significant association between maternal and newborn hemoglobin, underscoring the importance of preventing and correcting maternal anemia in pregnancy. Furthermore, maternal anemia should be considered a risk factor for neonatal anemia.

## 1. Background

Maternal anemia in pregnancy is defined as a hemoglobin (Hb) level <11 g/dl [1]. The global prevalence of maternal anemia in pregnancy is approximately 42% [2]. In Uganda, the prevalence has been estimated at 22%–33% [3, 4], although a recent study in Uganda reported a very high prevalence of 63% among pregnant women attending antenatal care at the Mbarara Regional Referral Hospital [5]. Maternal anemia can be due to blood loss, infection, and other causes, but nutritional deficiency is the most common cause globally [6]. Inadequate iron intake and poor infrastructure services for early diagnosis and treatment of anemia at the health facility level in resource-limited settings [7] contribute to anemia, and pregnant women are one of the most vulnerable groups [2]. Iron needs increase exponentially during pregnancy to meet the demands of the feto-placental unit, expand maternal erythrocyte mass, and compensate for iron loss through blood during delivery [2, 8].

Iron deficiency anemia in pregnancy may also affect the hemoglobin and iron reserves in the offspring, leading to newborn anemia [9, 10]. Anemia in newborns can be life-threatening [11]; causing delays in brain maturation, tissue hypoxia, arrested growth, and poorer cognitive, motor, and social-emotional development [12–14]. The proportion of newborn infants with anemia in sub-Saharan Africa ranges from 23% to 66% [15, 16]. One study found an overall positive correlation between maternal and umbilical cord hemoglobin (as a marker of newborn anemia), with lower umbilical cord blood hemoglobin levels related to anemic mothers [6].

However, there is a paucity of data on the strength of the correlation between maternal and newborn anemia, overall prevalence, and maternal risk factors for neonatal anemia in resource-limited settings. Such data are urgently needed to institute early interventions to reduce anemia-associated complications. Specifically, in Uganda, despite a number of published studies on the prevalence of maternal anemia, there is a dearth of information regarding the prevalence and risk factors for newborn anemia. To address this knowledge gap and contribute to the development of public health guidelines, we designed a prospective cohort substudy to assess the prevalence, correlation between cord blood and maternal anemia, and risk factors for newborn anemia in southwestern Uganda.

## 2. Methods

### 2.1. Participant Recruitment and Ethics

This was a cross-sectional study of 352 pregnant women presenting to the Mbarara Regional Referral Hospital (MRRH) for delivery. It was a substudy taken from a larger prospective cohort study of 600 pregnant women delivering their babies at MRRH and the 600 offspring born to those women. The hospital has a total bed capacity of 420 beds and serves a largely semiurban population. The study population consisted of 176 women living with HIV (WLWH) and 176 HIV-uninfected women and babies born to them. All women ≥18 years of age presenting in labor for delivery were eligible for enrolment. Women were excluded from the study if they did not speak English or Runyankole (the local language) well enough to give informed consent, had known or suspected multiple gestations, could not be reached by telephone after discharge for follow-up, were WLWH but not taking antiretroviral therapy (ART), or the study team was unable to collect the participant's placenta. All participants recruited in the cross-sectional study were included in the planned anemia analysis.

### 2.2. Ethical Approval

The study was approved by the institutional ethics review board at the Mbarara University of Science and Technology (MUST, 11/03-17), Partners Healthcare (2017P001319/MGH), and the Uganda National Council of Science and Technology (HS/2255). All participants gave written consent to participate, including consent to review the medical records of the woman and her baby for clinical history, treatment received, and outcome measures.

### 2.3. Sample Collection and Hemoglobin Determination

After written informed consent was obtained, maternal blood was collected via peripheral venipuncture. At delivery, the placenta was collected, and umbilical cord blood was obtained via venipuncture of the placental vein within 30 minutes of placental delivery. One drop of blood was used for hemoglobin determination using the point-of-care HemoCue Hb 301 according to the manufacturer's instructions (HemoCue, USA). Umbilical cord hemoglobin (Hb) was used as a proxy for newborn hemoglobin and newborns were classified as having polycythemia (Hb > 20 g/dl), normal (13–20 g/dl), and anemia (<13 g/dl). The primary outcome variable was umbilical cord hemoglobin level less than 13 g/dl. Maternal anemia was defined as Hb < 11 g/dl [4, 17].

### 2.4. Sample Size and Statistical Analysis

The sample size of 352 participants was selected to achieve sufficient power for the cross-sectional study aimed at ascertaining the prevalence and factors associated with newborn anemia. Summary statistics were used to characterize the cohort. Demographic characteristics and outcomes were compared between umbilical cord blood hemoglobin below 13 g/dl and 13 g/dl or more using chi-squared analysis for categorical variables and Student's *t*-test or Wilcoxon rank sum for continuous variables. Variables with *P* value <0.2 in association with newborn anemia were selected for the multivariable analysis. We then estimated the relationship between maternal and umbilical cord hemoglobin concentrations using linear regression analysis, adjusting for potential confounders. Predictor variables were selected using the *P* value screen on bivariate analysis as above and based on published associations, including maternal age, parity, employment, maternal HIV serostatus, maternal self-reported malaria diagnosis in pregnancy, mode of delivery, number of antenatal care visits, maternal report of deworming during pregnancy, maternal report of anemia diagnosed during pregnancy, diagnosis of antepartum hemorrhage (APH), maternal report of intake of ferrous iron/folic acid intake during pregnancy, and educational status. All variables with *P* value <0.05 in the final multivariate linear regression model were considered significant independent predictors of the outcome of newborn anemia. All analyses were performed using Stata software (Version 16.0, StataCorp, College Station, TX).

## 3. Results

Of the 352 maternal participants recruited, 60/352 had umbilical cord hemoglobin levels <13 g/dl, representing a prevalence of newborn anemia of 17% (Table 1). Of the 352 participants, 281 (79.8%) had a normal hemoglobin level while 11 (3.2%) had polycythemia. The mean age in years (SD) was significantly lower for participants with umbilical cord hemoglobin <13 g/dl than those ≥13 g/dl (26 [5.6] versus 28 [6.3], *P*=0.01). The mean birthweight did not differ significantly between participants with umbilical cord hemoglobin <13 g/dl and those with ≥13 g/dl (3.1 versus 3.2 kilograms, *P*=0.95, Table 1). There was no significant difference between the two groups in terms of maternal and newborn profiles except that the median parity for the anemic participants was 3, significantly higher than that of nonanemic participants which was 2 (*P*=0.01, Table 1).

**Figure 1:**
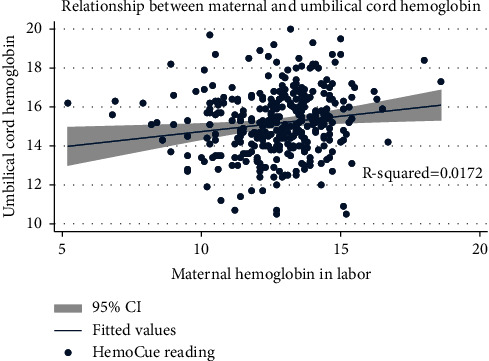
Scatter plot showing mean hemoglobin concentration of maternal and umbilical cord blood.

There was a significant positive correlation between maternal and umbilical cord hemoglobin concentrations (Figure 1, *R*^2^ = 0.017).

In multivariable linear regression analysis, for every one-point increase in maternal hemoglobin, there was a corresponding 0.15 point (unadjusted)/0.14 point (adjusted) increase in umbilical cord hemoglobin, and the result was statistically significant (*P*=0.02, Table 2). For every one-unit increase in maternal parity, there was a 0.15 point (unadjusted)/0.25 point (adjusted) decrease in umbilical cord hemoglobin, and the association was statistically significant (*P* ≤ 0.01). Cesarean delivery mode was also associated with a 0.39 point (unadjusted)/0.46 point (adjusted) decrease in umbilical cord hemoglobin level (*P*=0.03, Table 2). The lowest asset index quartile was significantly associated with decreased hemoglobin (*P*=0.03), indicating that lower socioeconomic status was associated with anemia. Maternal reports of receiving iron and/or folate in pregnancy, deworming in pregnancy, diagnosis of malaria in pregnancy, and number of antenatal care visits during pregnancy were not significantly associated with anemia in adjusted or unadjusted analyses.

## 4. Discussion

The prevalence of newborn anemia in this study was 17%, and there was a significant positive correlation between maternal and umbilical cord hemoglobin concentrations. The prevalence was lower than the prevalence reported in previous cross-sectional studies. For example, prior studies in Ethiopia reported a prevalence of newborn anemia ranging from 23% to 25% [15, 18]. However, these studies were conducted in a largely rural setting compared to our semiurban study population. Prior studies in resource-limited settings have noted that anemia is more prevalent in rural than urban and semiurban populations [19], which may partially explain these differing findings. In contrast, the prevalence of anemia in our populations was higher than what has been reported in the USA, Nepal, and Ethiopia which reported prevalence rates of 14%, 6%, and 9%, respectively [20–22]. These differences in anemia prevalence could be attributed to differences in socioeconomic conditions and clinical characteristics of the study participants. In addition, the differences could also be because in some previous studies, specifically [20], women with only iron deficiency anemia (IDA) were included, whereas our study included anemia of any cause.

We found a significant positive correlation between maternal and umbilical cord hemoglobin concentrations. For every 1-point increase in maternal hemoglobin, there was a corresponding 0.14-point increase in umbilical cord hemoglobin. The finding of a positive correlation between maternal and umbilical cord hemoglobin in this study is similar to other studies in Nepal, Kenya, Ethiopia, Israel, India, and Iran [22–25]. Since the fetus obtains iron from maternal transferrin, when maternal iron stores are depleted, the fetus cannot accumulate as much iron, resulting in a decrease in fetal iron stores and reduced fetal hemoglobin levels [10, 26–29]. The physiological changes and metabolic demands of pregnancy result in an increased requirement for iron in pregnancy [30], predisposing to maternal anemia. Maternal anemia leads to adaptations in placental and fetal physiology, resulting in pregnancy and birth complications such as low birth weight, neurodevelopmental disorders, and premature delivery [31, 32].

We also found a statistically significant association between parity and umbilical cord hemoglobin levels, with a 0.25-point decrease in umbilical cord hemoglobin for every one-unit increase in parity (*P* < 0.01). Higher parity increases the risk of developing iron deficiency anemia in pregnancy, and the incidence of anemia has been shown to increase with the number of pregnancies [33–36]. This association could be attributed to the depletion of maternal iron stores with each subsequent pregnancy or to inadequate spacing between pregnancies that often comes with high parity and is common among Ugandan mothers [37–39]. The etiology of low hemoglobin in a newborn is multifactorial, with prenatal factors like maternal malnutrition, iron deficiency anemia, and infections being the most common [40].

In addition, we found that cesarean delivery was significantly associated with a 0.46-point decrease in umbilical cord hemoglobin level. Cesarean delivery has previously been described to increase the risk of postpartum anemia by two-fold [26], due to the increased risk of uterine atony and severed vessels when the abdominal wall is opened [41, 42]. Lastly, we found that lower wealth, measured using the asset index, was associated with lower umbilical cord hemoglobin levels. These findings are similar to those in other settings [43], although a study in Indonesia found no direct relationship between socioeconomic status and anemia [44]. Women with lower socioeconomic status may have limited access to nutritious food, which can lead to poor maternal nutritional status and subsequent fetal anemia [45]. We found a significant positive correlation between maternal and newborn hemoglobin levels. Parity, cesarean delivery, and lower asset index quartiles were significantly associated with newborn anemia. Such information is required to institute early interventions to reduce anemia-associated complications. This underscores the importance of preventing maternal anemia and maintaining adequate iron stores during pregnancy.

## 5. Conclusions

The prevalence of newborn anemia in this study was 17%. We found a significant positive correlation between maternal and umbilical cord hemoglobin concentrations. We also found a statistically significant association between parity and umbilical cord hemoglobin levels, with a 0.25-point decrease in umbilical cord hemoglobin for every one-unit increase in parity. Cesarean delivery was significantly associated with a 0.46-point decrease in umbilical cord hemoglobin level.

Based on our results, we recommend that implementation of routine monitoring of maternal hemoglobin levels be strengthened as outlined in obstetric care policy guidelines [46]. In addition, umbilical cord hemoglobin may also be useful for early diagnosis and intervention for newborn anemia. A future study on predicting fetal anemia during pregnancy by measuring middle cerebral artery peak systolic velocity (MCA PSV) Doppler should be considered in this population of mothers. Efforts should also be made to improve the socioeconomic status of pregnant women and provide them with adequate access to antenatal care and nutritional support. Overall, ensuring adequate iron stores during pregnancy through good nutrition, iron supplementation, and spacing pregnancy appropriately are key policy strategies for preventing maternal anemia and its associated complications in sub-Saharan Africa and other resource-limited settings.

### 5.1. Limitation

Newborn anemia in mothers with haemoglobinopathies or thalassemia would be higher than other etiologies [47, 48]. We, however, did not ascertain the genetic etiologies of newborn anemia such as haemoglobinopathies and alloimmunization due to limited resources.

## Figures and Tables

**Table 1:** Sociodemographic characteristics and clinical data of study participants, compared by umbilical cord hemoglobin level.

Participant characteristics	Umbilical cord blood Hb ≥ 13 g/dl	Umbilical cord blood Hb < 13 g/dl	*P* value
Maternal characteristics			
Parity, median (IQR)	2 (1–4)	3 (2–5)	**0.01**
Married, *n* (%)	30 (90.1)	287 (90.0)	0.86
Age, mean (SD)	26 (5.6)	28 (6.3)	**0.01**
HIV-infected, *n* (%)	155 (48.6)	21 (63.6)	0.10
Resides in mbarara, *n* (%)	204 (64.2)	23 (70.0)	0.81
Formally employed, *n* (%)	118 (37.0)	9 (27.3)	0.27
Primary education or less, *n* (%)	185 (58.8)	22 (58.0)	0.34
Obstetric and self-reported characteristics			
Delivered by cesarean, *n* (%)	101 (32.4)	13 (39.4)	0.37
Attended ≥4 ANC visits, *n* (%)	200 (62.7)	23 (69.7)	0.43
Maternal self-report of no anemia diagnosed during pregnancy, *n* (%)	315 (98.8)	33 (100.0)	0.52
Maternal self-report of deworming during pregnancy, *n* (%)	273 (85.6)	28 (84.8)	0.91
Maternal self-report of iron/folate intake during pregnancy, *n* (%)	219 (68.7)	23 (69.7)	0.90
Maternal self-reported malaria diagnosis in pregnancy, *n* (%)	38 (11.9)	4 (12.1)	0.97
Neonatal characteristics			
Birthweight, mean (SD)	3.2 (0.4)	3.1 (0.5)	0.95

*Note*. Hb–hemoglobin; HIV–human immunodeficiency virus; ANC–antenatal care; SD–standard deviation. The bold values were to emphasize the variables where there was a significant *P*‐value difference of less than 0.05 between the two groups of participants i.e those with umbilical cord blood Hb ≥ 13 g/dl and umbilical cord blood Hb < 13 g/dl.

**Table 2:** Univariable and multivariable linear regression analysis of risk factors for newborn anemia.

Characteristic	Univariable		Multivariable	
	*β* coefficient (95% CI)	*P* value	*β* coefficient (95% CI)	*P* value
Maternal hemoglobin	0.15 (0.04–0.27)	0.01	0.14 (0.03–0.26)	**0.02**
Age	−0.03 (−0.06–0.01)	0.14	0.03 (−0.02–0.09)	0.22
Parity	−0.15 (−0.27–0.04)	0.01	−0.25 (−0.43–−0.07)	**<0.01**
Cesarean delivery	−0.39 (−0.81–0.26)	0.07	−0.46 (−0.88–−0.04)	**0.03**
Self-reported intake of iron/folate in pregnancy	−0.39 (−2.26–1.47)	0.68	−0.56 (−2.4–1.3)	0.56
Self-reported malaria in pregnancy	0.10 (−0.51–0.71)	0.74	1.03 (0.56, 1.88)	0.93
Attended antenatal care <4 times	0.24 (−0.17–0.65)	0.25	1.17 (0.78, 1.77)	0.45
Asset index quartile				
Poorest	−0.35 (−0.91–0.21)	0.22	−0.42 (−0.98–0.15)	0.15
Wealthier	−0.49 (−1.05–0.69)	0.09	−0.55 (−1.11–0.01)	0.06
Wealthiest	−0.38 (−0.94–0.18)	0.18	−0.65 (−1.23–−0.06)	**0.03**
Self-report of deworming during ANC	−0.21 (−0.35–0.77)	0.48	0.22 (−0.33–0.79)	0.42

*Note*. ANC–antenatal care; CI–confidential interval. The bold values were to emphasize the variables where there was a significant association for newborn anemia with a *P*‐value difference of less than 0.05 at multivariable analysis after controlling for confounders.

## Data Availability

The datasets used and analyzed during the current study are available from the corresponding author upon reasonable request.
